# Focal and Ambient Processing of Built Environments: Intellectual and Atmospheric Experiences of Architecture

**DOI:** 10.3389/fpsyg.2017.00326

**Published:** 2017-03-16

**Authors:** Kevin K. Rooney, Robert J. Condia, Lester C. Loschky

**Affiliations:** ^1^College of Architecture, Planning, and Design, Kansas State UniversityManhattan, KS, USA; ^2^Department of Architecture, Kansas State UniversityManhattan, KS, USA; ^3^Visual Cognition Lab, Department of Psychological Sciences, Kansas State UniversityManhattan, KS, USA

**Keywords:** focal attention, ambient processing, intellectual attention, atmospheric awareness, central visual field, peripheral visual field, architectural experience

## Abstract

Neuroscience has well established that human vision divides into the central and peripheral fields of view. Central vision extends from the point of gaze (where we are looking) out to about 5° of visual angle (the width of one’s fist at arm’s length), while peripheral vision is the vast remainder of the visual field. These visual fields project to the parvo and magno ganglion cells, which process distinctly different types of information from the world around us and project that information to the ventral and dorsal visual streams, respectively. Building on the dorsal/ventral stream dichotomy, we can further distinguish between focal processing of central vision, and ambient processing of peripheral vision. Thus, our visual processing of and attention to objects and scenes depends on how and where these stimuli fall on the retina. The built environment is no exception to these dependencies, specifically in terms of how focal object perception and ambient spatial perception create different types of experiences we have with built environments. We argue that these foundational mechanisms of the eye and the visual stream are limiting parameters of architectural experience. We hypothesize that people experience architecture in two basic ways based on these visual limitations; by intellectually assessing architecture consciously through focal object processing and assessing architecture in terms of atmosphere through pre-conscious ambient spatial processing. Furthermore, these separate ways of processing architectural stimuli operate in parallel throughout the visual perceptual system. Thus, a more comprehensive understanding of architecture must take into account that built environments are stimuli that are treated differently by focal and ambient vision, which enable intellectual analysis of architectural experience versus the experience of architectural atmosphere, respectively. We offer this theoretical model to help advance a more precise understanding of the experience of architecture, which can be tested through future experimentation. (298 words)

## Introduction

**FIGURE 1:**
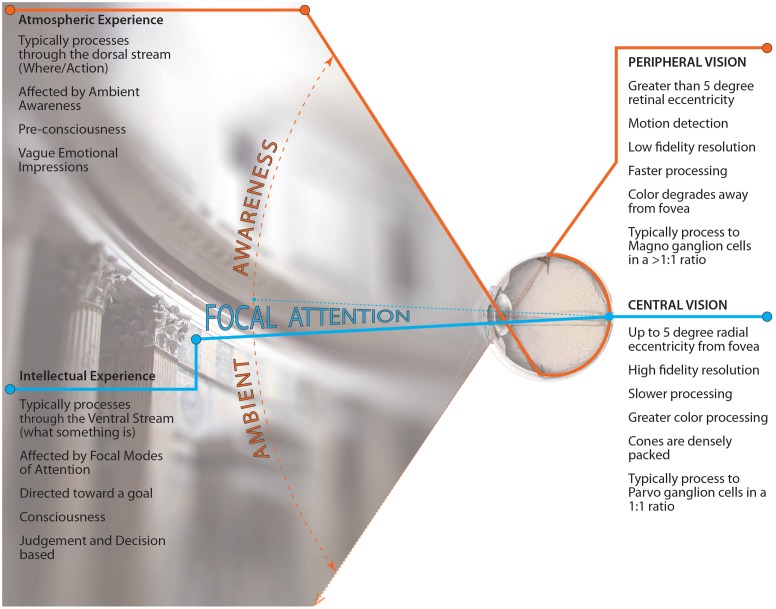
**Diagram of hypotheses**. Image shows the divided nature of visual perception and the overlay of architectural experience associated along each division of vision (i.e., the central visual field/focal vision associated with intellectual architectural experience and the peripheral visual field / ambient vision associated with atmospheric awareness of architecture). Image courtesy of Kevin Rooney.

Our hypothesis is that the visual experience of architecture is divided into two types based on the mechanisms of the eye and brain, which strongly affect our visual awareness and experience within architecture; see **Figure 1**. The first type of experience hypothesized is our intellectual attention to architecture (i.e., focally attending to particular aspects of the architecture) which yields descriptions of the foveated architectural details. The other type of experience is the ambient atmosphere of architecture (i.e., ambiently processing the overall mood of the space) which yields emotional responses to the architectural space. If our hypothesis is correct, then architecture allows for two different responses to the same architectural design based on its location in the visual field. However, because ambient processing is often outside of conscious awareness, the problem is that our understanding of peripheral visual processing as an essential feature of the architectural experience is underdeveloped.

It is important to note that our hypotheses rely on a model of a divided visual system, namely central and peripheral visual fields on through to the dorsal and ventral stream of vision (for reviews, see; Wilson et al., 1990; Previc, 1998; Strasburger et al., 2011; Whitney and Levi, 2011; Loschky et al., 2017). However, we do not claim that the human visual system is reductive to a strict dichotomy without crossover. Nevertheless, the general nature of a divided visual system discussed in our model is based on a large body of research that is mostly unchallenged and helps explain a great deal about visual sensation and perception. We will provide a necessarily very brief overview of the neurophysiological structures that support our model of a divided visual system and also the deployment of attention and awareness within the model (for more detailed coverage of the visual system, see Siegelbaum and Hudspeth, 2000, ch. 25–27; Palmer, 1999; Frisby and Stone, 2010). Finally, to bridge to a larger audience, including interested architects and others interested in architecture, we have provided a more descriptive review of the visual system to establish a broader foundation for those not familiar with the general model of the visual system, attention and awareness.

### The Retina as a Visual Field

**FIGURE 2:**
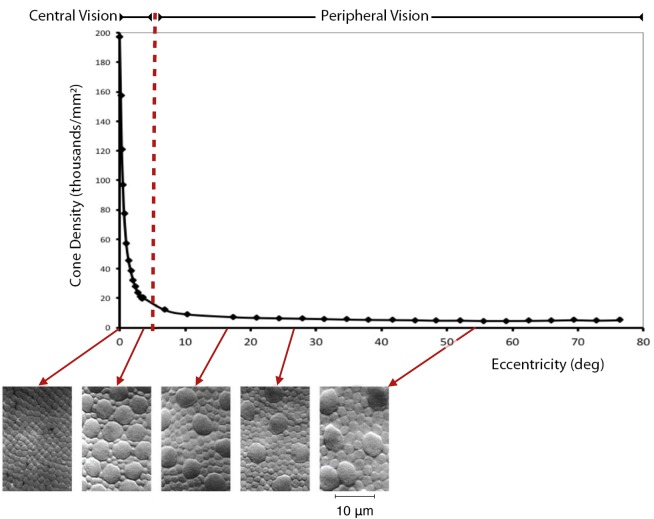
**Distribution of cones by retinal eccentricity**. The density of cone photoreceptors is shown to drop off exponentially with distance from the center of vision (0 degrees from the fovea). This results in the loss of visual resolution with increasing distance from the fovea. The upper image drawn from data digitized from Curcio et al. (1990, Figures 6a,c) and averaged over nasal and temporal directions (with the gap at the blind spot ignored). Cone density given in # cells/degrees 2 × 1000. Degrees estimated from mm using 0.288 deg/mm from Drasdo and Fowler (1974). Lower micrographs from Curcio et al. (1990, Figures 2, 3). All Curcio et al. images reproduced with the permission of John Wiley and Sons.

Light passes through the lens allowing it to project onto the back wall of the eye called the retina. The retina has a network of about 100 million photoreceptors are divided into two types; cones and rods. There are two important issues regarding cones that impact the argument we are making regarding the visual experience of architecture. The first issue is that the distribution of cones is highly concentrated at the center of vision, the fovea (see **Figure 2**). The density of cones is highest in the fovea, and drops off exponentially with increasing distance from it. Given the high density of cones in the central visual field, visual details (encoded by higher spatial frequencies) are best seen with central vision (Livingstone and Hubel, 1988).

This concentration allows for higher acuity in both form and color in and around the fovea, called central vision, which encompasses approximately the central 5° radius from the center of the fovea. Central vision predominates our conscious vision, and contains what we consider ourselves to be looking at. Beyond 5° of visual eccentricity, which is referred to as peripheral vision, cone density quickly decreases and levels off for the remainder of our entire visual field. The peripheral visual field predominates our pre-conscious vision from which we come to know our visual place in the world (Leibowitz and Post, 1982; Previc, 1998).

The second major issue is that ganglion cells, which send information from the retina to the brain, pool information from cones differentially between central and peripheral vision. In peripheral vision, a single magno ganglion cell (M cell) will typically connect to many cone receptors. This convergence reduces visual resolution through aggregation. On the other hand, M cells are larger and have thicker myelin sheaths, and thus have higher processing speeds. The M cells are therefore better at conveying information useful in detecting motion, which plays an important role in spatial perception. Conversely, in central vision, a single parvo ganglion cell (P cell) typically connects to a single cone receptor. This lack of convergence produces higher visual resolution of detail. On the other hand, P cells are smaller, with thinner myelin sheaths, and thus have lower processing speeds compared to M cells. P cells are therefore better at conveying information useful for detecting differences in color and form, which are very important for clearly identifying objects, but worse at detecting motion. The M and P cells transfer their output to the subcortical structure of the lateral geniculate nucleus (LGN), which further amplifies the differences between central and peripheral vision. The LGN then passes its output to the primary visual cortex (aka, V1) at the back of the brain, where there are many more cells devoted to central vision (the central 5° radius of vision), which is known as cortical magnification of fovea.

Beyond V1, information is separated along the dorsal and ventral visual streams. The ventral stream is generally understood to process what things are (Mishkin et al., 1983) and our conscious perception of objects and their colors and shapes (Goodale and Milner, 1992). The dorsal stream is generally understood to process where things are (Mishkin et al., 1983) and enables us to interact with things and our spatial environment (e.g., manipulating objects, navigation, etc.) (Goodale and Milner, 1992). For simplicity, and consistent with common usage, we will call the ventral stream the “what pathway” and the dorsal stream the “where pathway” (Mishkin et al., 1983). Importantly, the transformations of visual information from the retina to V1 are passed along to the what and where pathways, which show biases towards central and peripheral vision, respectively. The central visual field greatly supports the processes underlying the conscious perception of objects in the what pathway. For example, the lateral occipital area (LOC), which is in the what pathway and is greatly involved in object recognition, has a strong central vision bias (Grill-Spector et al., 2001; Larsson and Heeger, 2006). The peripheral visual field greatly supports the processing of spatial relations, actions on objects, and navigation of the environment in the where pathway. For example, area V6, which is in the dorsal stream, and processes motion information used to navigate (known as optic flow), exclusively processes information from the visual periphery (Pitzalis et al., 2010, 2013). Nevertheless, there is a visual area in the what pathway that has a strong peripheral vision bias, namely the Parahippocampal Place Area (PPA), which responds to places more strongly than to objects, people, or faces (Malach et al., 2002; Arcaro et al., 2009; Nasr et al., 2011). Thus, even in this case, peripheral vision is associated with processing the big picture of the environment, rather than objects. Conversely, areas of the parietal cortex in the where pathway are strongly involved in grasping things with the hands (Goodale and Milner, 1992), which crucially involves central vision–we look at things before grasping them, to guide fine hand movements. Thus, even in this case, central vision is associated with processing details and objects. These latter examples show that rather than there being a perfect division of central vision to the what pathway, and peripheral vision to the where pathway, central vision is devoted to detailed perception of objects and their forms, colors and shapes, and peripheral vision is devoted to coarse perception of our environment, our place in it, and movement through it.

The above discussion of the anatomical and functional specializations of central and peripheral vision, from the retina to the what and where pathways, lays the foundation for understanding the different roles of central and peripheral vision. Those functional distinctions map on quite well to what are called the focal and ambient modes of vision (Leibowitz and Post, 1982). In a nutshell, the focal mode involves the use of central vision to scrutinize objects, whether distant or near, and our hands as we interact with them (Leibowitz and Post, 1982; Previc, 1998). To those previously proposed functions, we would add that focal mode generally concerns things we are paying attention to because we are looking at them. The ambient mode involves the use of our entire visual field, which is primarily in peripheral vision, in order to maintain our balance, and navigate through our environment (Leibowitz and Post, 1982; Previc, 1998). To those previously proposed functions, we would add that the ambient mode also lets us roughly know what our surroundings are (i.e., scene gist), and allows the brain to decide (usually unconsciously) what we should pay attention to next. We will argue in the remainder of our paper that the focal and ambient modes are particularly important for our experience and appreciation of architecture. We will argue that a built environment can be perceived in central vision, in the focal mode, as an object (or collection of objects), or in peripheral vision, in the ambient mode, as a space to be in or move through. Furthermore, we will argue that our level of conscious awareness, and intellectual engagement with architecture is primarily experienced through central vision in the focal mode. Conversely, we will argue that our gut reactions to a built environment, and our experience (often unconscious) of its atmosphere, are primarily perceived through peripheral vision in the ambient mode.

## Simple Objects and Simple Scenes

In this section, we review how the two fields of vision analyze and perceive stimuli in the world in order to show which features are important to which type of architectural stimulus. First, we will review object perception and its greater dependence on the *focal mode* of processing through central vision in the *what* pathway. Then we will discuss scene perception and with its greater dependency on the *ambient mode* of visual processing through peripheral vision in the *where* pathway.

What is the difference between objects and scenes? For the sake of this section, objects will refer to those things being seen as individual, or countable as a single unit in-and-of-themselves when looked at; a coffee cup for example. Scenes, on the other hand, are the visual perception of a place we are looking onto; a beach for example. For the most part, we will argue that objects, like cups, are best recognized when seen in the central visual field, whereas scenes, like beaches, are best recognized when seen in the peripheral visual field.

### Object Perception

**FIGURE 3:**
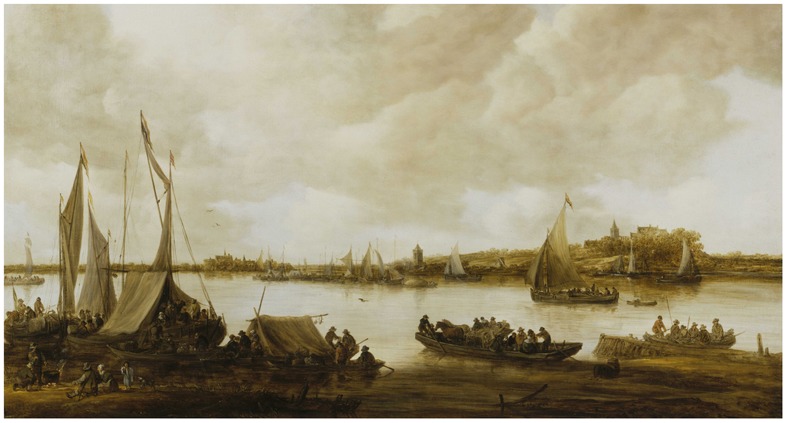
**Jan van Goyen’s view of the Rhine near Hochelten (1653)**. The painting is composed of painted objects which depict a river scene. Of most interest is the blackbird flying near the lower center of the painting. The first author (KKR) notes that when seeing it in person, by staring at the blackbird, the scene takes on a better illusion of depth. http://ww2.hdnux.com/photos/17/12/72/3976885/3/628x471.jpg.

At the outset of discussing what an object is and how it is perceived, consider the example of Jan van Goyen’s painting, View of the Rhine near Hochelten (1653), shown in **Figure 3**. In the painting, we see many objects; several different types of boats, a couple of tents, and a number of people. This leads to an interesting question, how do we see objects in paintings, or in the real world? In the previous section, we discussed how the *what* pathway, which is heavily involved in object recognition, makes great use of the central visual field, which has higher resolution than peripheral vision. This higher resolution contains higher spatial frequencies, which are useful for perceiving the edges of a form, which in turn are critically important for recognizing objects (Biederman, 1987). Thus, paintings work because they exploit the way our visual system operates. For recognizing objects, paintings, and even more so line drawings, provide the high spatial frequencies necessary for seeing edges, that the ventral visual stream uses to recognize objects such as boats, tents, and people.

**FIGURE 4:**
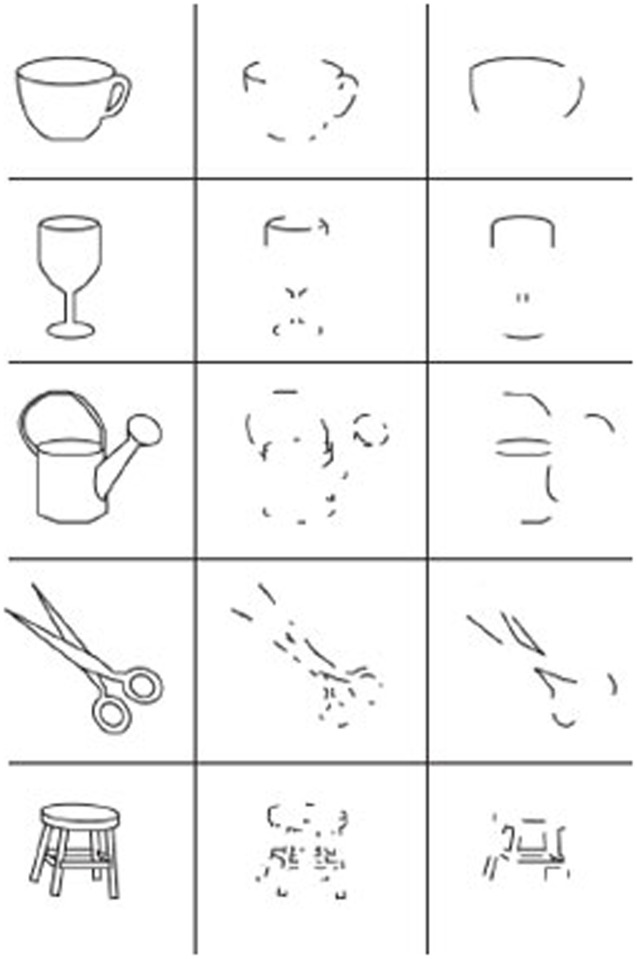
**Biederman’s RBC images**. Example of five stimulus objects in the experiment on the perception of degraded objects. (The left column shows the original versions. The middle column shows the recoverable versions. The contours have been deleted in regions where they can be replaced through collinearity or smooth curvature. The right column shows the non-recoverable versions. The contours have been deleted at regions of concavity so that collinearity or smooth curvature of the segments bridges the concavity. In addition, vertices have been altered, for example, from Ys to Ls, and misleading symmetry and parallelism have been introduced) (Biederman, 1987). Image reproduced with the permission of American Psychological Association.

Irving Biederman presents a theory of how we recognize objects in the world in his classic model called Recognition by Components (RBC). Biederman’s (1987) RBC theory argues that object recognition critically depends on the edges and vertices of an object. Specifically, he argues that “T”, “Y” and “↑” vertices, formed by edge contrasts, provide the critical information needed to determine the fundamental shapes that make up an object, as shown in the middle column of images in **Figure 4**.

In order to clearly see such edge distinctions to recognize an object, we must look at it, which involves pointing our fovea (central vision) at the object. It is much more difficult to recognize objects in our peripheral visual field, because vision there is lower resolution, and becomes increasingly so with greater eccentricity. As noted earlier, this is due to the increasingly aggregated retinal processing by the M ganglion cells (Wilkinson et al., 2016), thus making peripheral vision much worse than central vision for recognizing objects (Livingstone and Hubel, 1988; Strasburger et al., 2011; Boucart et al., 2016). Biederman’s theory explains how our ability to see the finer details of edges and their vertices allows us to recognize what the object is that we are looking at.

In addition, there is a further difficulty in recognizing objects in peripheral vision, which is caused by what is known as *crowding* (Bouma, 1970; Pelli and Tillman, 2008; Strasburger et al., 2011; Whitney and Levi, 2011). That is, it becomes difficult to recognize objects in peripheral vision if there are flanking objects near the target object (the one you are trying to recognize). More specifically, if the distance between a flanking object and the target object is less than half the distance of the target object to the center of the fovea, the target object will likely suffer crowding, a pattern of results known as Bouma’s law (Bouma, 1970). Furthermore, while most research on crowding has used simple stimuli (e.g., letters or Gabor patches), a recent study has shown that crowding also occurs for objects in real-world scenes (actually, computer-generated architectural models of scenes) (Coy et al., 2014).

**FIGURE 5:**
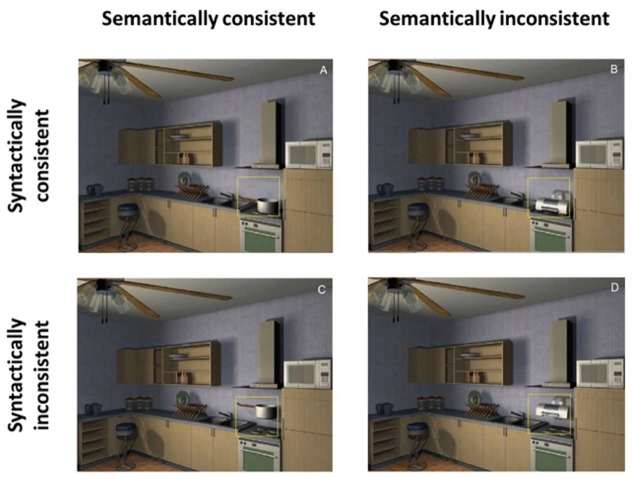
**Võ and Henderson’s (2011) study**. Consistent and inconsistent objects within a kitchen scene. The printer went without detection when viewed in the peripheral visual field. Image reproduced with the permission of Springer International Publishing AG.

Based on the above, there is an abundant research showing that object recognition is worse in peripheral vision than central vision (Boucart et al., 2016; Ehinger and Rosenholtz, 2016; for review, see Strasburger et al., 2011). Interestingly, not only is object recognition degraded in the visual periphery, but so too is memory for what is seen with peripheral vision (Geringswald et al., 2016), and this seems to be due to peripheral vision also having worse spatial coherence (i.e., degraded structure), which is consistent with theories of crowding in peripheral vision (Velisavljević and Elder, 2008). Furthermore, when an object seems odd in its surroundings, namely it is semantically inconsistent, like a printer sitting on a stove, when viewed with our peripheral vision it does not immediately grab our attention (Võ and Henderson, 2011). This suggests the peripheral visual system is poor at detecting that objects are inconsistent within the rest of the scene they are in; see **Figure 5**. Instead, objects in the periphery of a scene may provide more global characteristics of that scene rather than specifics (Eberhardt et al., 2016; Ehinger and Rosenholtz, 2016). In sum, there is considerable evidence that we are best able to consciously understand an object when we look at it (fixate it, or foveate it) with our central vision.

### Window vs. Scotoma Conditions

**FIGURE 6:**
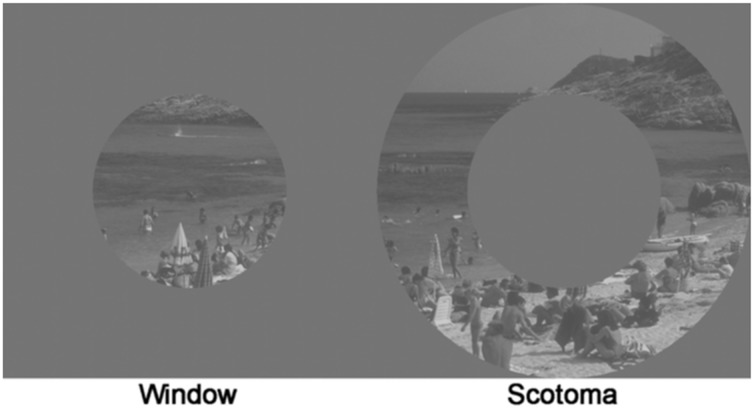
**Window/Scotoma conditions**. Window condition shown on left, while Scotoma condition is shown on right (Larson et al., 2014). Image reproduced with the permission of American Psychological Association.

Another way to explore the roles of central and peripheral vision is through using so-called window and scotoma conditions. Imagine an experiment in which we can block out either your central visual field, which we will call the *scotoma* condition, or your peripheral visual field, which we will call the *window* condition. Window and scotoma conditions are useful in dividing visual input between central and peripheral vision. As shown in **Figure 6**, an example window condition would be a circular region showing the central 5° radius of vision, and replacing everything outside that central *window* with neutral gray, thus only allowing you to see what is in your central visual field. Conversely, the inverse of the window is the *scotoma* condition, which replaces everything in the central 5° radius of vision with neutral gray (the *scotoma*), but shows everything outside that. Thus, the scotoma condition only allows you to see what is in your peripheral visual field (Larson and Loschky, 2009; Larson et al., 2014). If, under the scotoma condition, you tried looking at a cup on the table you would find it nearly impossible to figure out whether it was a cup of coffee, tea, or hot chocolate. This is the real-life problem faced by people afflicted by central scotomas caused by age-related macular degradation (a retinal disease of the fovea). If that blindness was reversed by being in the window condition, you would have a lot less trouble distinguishing what was in the cup, but you have problems interacting with the cup, such as reaching for it, because you would be blinded to the rest of the scene in your visual periphery. This is the problem faced by those afflicted by glaucoma and retinitis pigmentosa (retinal diseases of the visual periphery). Studies of the roles of central and peripheral vision on scene perception have frequently used such experimental window and scotoma conditions.

### Scene Perception

If, instead of wanting to recognize and inspect an object, we wanted to understand the place, or scene, we are located in, we will need to use our peripheral vision. Returning to Jan van Goyen’s painting of the Rhein, we may easily say, even in the absence of its title, that it is a river scene, which we can call its scene “gist.” Interestingly, if we saw this painting in the scotoma condition, namely without central vision, our sense of it being a river scene would likely remain unchanged. Larson and Loschky’s (2009) scene gist study, which utilized window and scotoma conditions, they found that the accuracy of scene gist identification with a 5° radius Scotoma was no worse than when seeing the entire scene. Conversely, scene gist identification with a 5° radius Window was considerably worse than seeing the entire scene. Together these findings suggested that peripheral vision was more important for understanding what a scene is than central vision. In a more recent study, Loschky et al. (2015) used similar methods with panoramic views of scenes (180° horizontally × 40° vertically), and found similar results for 5° radius Window and Scotoma conditions. Other research has shown that peripheral vision is also very useful for identifying the location of a scene (Eberhardt et al., 2016).

The above experiments suggest that the peripheral visual field provides most of the important information for recognizing what a scene is, namely its gist, and where the scene is located. Furthermore, it seems likely that this dominance of peripheral vision for recognizing scenes is due in part to the fact that our peripheral visual field is so huge (thus full of information), but also due to the ways in which this information is processed by the brain. The features most important to scene recognition are the same features the M ganglion cells are best at processing in the peripheral visual field. The aggregate nature of information along this pathway amplifies the visibility of larger contrasts and motion (perceived or real) resulting in a bias toward lower spatial frequencies (blurry blobs), which is one of the main features accounting for the perception of a scene. Peripheral vision is limited to processing lower spatial frequencies, and configurations of low spatial frequency blobs are very useful in recognizing scene gist (Schyns and Oliva, 1994). Thus, the work described above suggests that peripheral vision plays a critically important role in perceiving what scenes are and where they are due the amount of visible area in a scene and the analysis of low spatial frequency configurations (i.e., blobby layouts). This is consistent with findings that the brain area known as the PPA, which is strongly involved in recognizing the gist of scenes (Walther et al., 2009), shows a strong peripheral vision bias (Arcaro et al., 2009; Baldassano et al., 2016).

## Awareness and Attention

Based on what we know about visual processing from the retina through the *what* and *where* pathways, the division of the visual field between central and peripheral vision suggests a distinction between perception of stimuli in the world in terms of objects versus scenes. But what is meant by the word *perception*? Our discussion so far has concerned how visual information is filtered and divided, but has not addressed how we come to be *aware* of that information. If I look at Goyen’s painting showing a river scene, and my eyes fixate the boats, tents, and people, then can we say that I must surely be consciously aware of all those things? How about walking along in a similar scene in the real world, and looking at (fixating) similar objects? The answers to those simple questions are quite complex, because awareness is also not a simple singular process. Awareness too has its own divisions of focal and ambient processing which interact with the mechanisms of the eye either consciously or pre-consciously (Bánréti-Fuchs, 1967; Leibowitz and Post, 1982; Merikle et al., 2001; Horrey and Wickens, 2004). In this final section prior to integrating architecture into the conversation, we will describe the nature of awareness and attentional allocation and we will give some important everyday examples of how awareness is achieved.

### Focal and Ambient Processing and Awareness

How does awareness operate when we walk, drive, or, for that matter, when we stand? In Leibowitz and Post’s work on ambient processing, they described how even the act of standing utilizes our visual processing of the world (1982). According to Leibowitz and Post (1982), when we are standing, our visual processing of features in the world connects with other senses (e.g., the vestibular and kinesthetic senses) to enable us to maintain our balance and our position among the objects surrounding us. They describe the ambient mode of vision as pre-conscious and dependent on the “coordination of motor activity with the visual, vestibular, auditory and somatosensory systems, particularly, kinesthesis” (Leibowitz and Post, 1982, p. 344). In other words, the ambient mode is the coordination of our senses, including vision, to aid in determining our place in the world rather than our conscious analysis of it.

The ambient mode of vision is concerned with earth-fixed space, and spatial orientation and postural control in locomotion (Horrey and Wickens, 2004). It encompasses 180° of frontal vision and is dominantly influenced by the lower visual field, due to using the ground-based optic flow (e.g., what you see in the classic Windows “star field” screensaver) for walking. Visual ambient processing accesses information from great distances, relies on earth-fixed coordinate systems, and arm, leg, and head movements are the primary movements it guides. Important cues are the horizon, linear perspective, and optic flow. For navigating through an environment, fine detailed information is often less important than its global structure, and so peripheral vision becomes very important (Turano et al., 2005; Franchak and Adolph, 2010; Barton et al., 2014; Ehinger and Rosenholtz, 2016). The ambient mode of vision is typically not processed consciously and is possible even with low levels of luminance and decreased image quality such as low spatial frequencies (blurry blobs).

When walking, we use both central and peripheral vision, but much of the visual processing needed can be handled by ambient processing of peripheral vision. For walking, central vision is mostly used for determining the location of one’s goal. Thus, when walking, people normally look straight ahead or fixate their goal to navigate to it (Turano et al., 2001). However, when patients with peripheral vision loss walk, they frequently fixate the ground in front of them or walls to gather information on the layout of the scene, to situate themselves within their visual environment—people with normal vision use their peripheral vision for those functions. Thus, while walking through a space, people use their peripheral vision to create a coherent mental representation of the spatial relationships between objects in a space (Fortenbaugh et al., 2007, 2008). Specifically, having either a real or simulated peripheral vision loss (e.g., a moving scotoma) produces faulty mental representations of a space (producing misremembered locations of objects). Furthermore, when walking, normally sighted people primarily use their peripheral vision to alter their course or their gait to avoid obstacles—people rarely fixate such obstacles before walking over or onto them (only 20–30% of the time) (Marigold et al., 2007; Franchak and Adolph, 2010). An extreme example in the real world is the fact that it is possible to walk, albeit more slowly and hesitatingly, while texting (Plummer et al., 2015)—note that while this is dangerous, because it almost surely increases the likelihood of collisions, it illustrates the use of ambient vision in walking.

Similarly, when considering the everyday act of driving, studies have shown that a driver can maintain their lane position while looking at and paying close attention to information on the car dashboard (Summala et al., 1996; Horrey and Wickens, 2004). Conversely, ambient vision was not sufficient to detect hazardous, or unexpected situations, such as a lead vehicle suddenly braking (Summala et al., 1998; Lamble et al., 1999; Horrey et al., 2006). So, as we are walking or driving and daydreaming, it is our pre-conscious ambient visual perception which keeps us on our path or on the road, but unfortunately does not help much in seeing the child between two signs about to dart out in front of us.

To see a child about to enter the road, we often must utilize our central visual field and attend to them with what Leibowitz and Post (1982) refer to as focal attention. Horrey and Wickens (2004) suggest that focal attention operates within the central 20°–30° of the upper visual field. Focal attention also spans from 0.2 m (8 inches) to great distances, relies on eye movements (saccades) as the primary motor system, and is centered in the retinotopic coordinate system (i.e., what is seen at any given moment, and its position on the retina). It is also highly represented in conscious awareness and is adversely affected by low levels of luminance, decreased image quality, and lower spatial frequencies. Thus, focal attention plays a critical role in our conscious awareness of objects in our surroundings.

### Attentional Allocation and Deployment

Visual attention has been studied in great depth over the past 50 years (for reviews, see Pashler, 1998; Carrasco, 2011; Chun et al., 2011). There are two basic manners in which we visually attend to the world. We can attend to something overtly by looking at it (i.e., fixating it with our eyes; e.g., looking to the left after hearing glass break) or we can attend to something covertly without actually looking at it (e.g., fixating our target in war ball but attending to their teammate to their right without moving our eyes). Importantly, paying attention to something, even covertly (i.e., without fixating it), can improve your perception of it, allowing you to perceive more details in it (Carrasco, 2011), and allowing it to enter your conscious awareness (Kuhn and Findlay, 2009).

There are also different ways that our brains filter out the overwhelming amount of visual information contained in the world by attending to some manageably small amount of it at a given moment, which are known as spatial, feature-based, and object-based attentional selection. Spatial attentional selection can be understood as where in our visual field we attend to at a given moment. For example, when searching for your car in a parking lot, at a given moment, you might attend only to the location you are fixating, or you might broaden your attention to encompass your entire visual field, including all of the parking lot you can see at that moment. Feature-based attentional selection involves focusing attention on certain features over others, such as if your car is red, then not attending to all of the cars in the parking lot, but instead only attending to the red ones. Object-based attentional selection involves focusing attention on entire objects, whatever their shape, size, color, or location in your visual field; for example, when you finally find your car, attending only to it, rather than some particular location containing three red cars, including yours and two others. You can intentionally deploy your attention, known as endogenous deployment, for example looking both ways before crossing a street. Or your attention can be captured by an unexpected stimulus, such as a blinking light, known as exogenous deployment. Additionally, as suggested above, when discussing spatial selective attention, you can focus your attention narrowly, like a spotlight (Posner, 1980) or spread it out broadly, like a zoom lens (Eriksen and Yeh, 1985). The effect of either focusing or spreading out your attention is similar to the costs and benefits of central and peripheral vision. The more spread out your attention is, the more things you can consciously perceive, but the fewer details you are able to perceive of each thing. The more narrowly focused you attend, the better you can see what you are attending to, but the more blinded you become to the things you are ignoring (Ikeda and Takeuchi, 1975; Williams, 1989; Ringer et al., 2016).

Importantly, the benefits and costs of visual attention on perception are subject to the hard-visual limitations set by the eye and brain in the central and peripheral visual fields. Thus, if you attend to something covertly in your visual periphery, you will perceive it better than if you had ignored it, but you may still find it difficult to determine what it is, for all of the reasons we have discussed earlier (i.e., low resolution and crowding in peripheral vision, due to the physiology of the retina, and visual processing by the brain).

Attention and awareness are also important to the distinction between focal and ambient processing. For example, we mentioned earlier that drivers can maintain their lane position even when their attention is focused below the dashboard on their radio (Summala et al., 1996). This suggests that ambient processing of peripheral vision, which has been argued to be pre-conscious (Previc, 1998), is unaffected by focusing attention, and awareness, in central vision. However, later research by Horrey et al. (2006) has shown that this is not completely true. They found that drivers’ lane-keeping performance using peripheral vision was somewhat degraded both as more time was spent looking at a visual display below the dashboard, and also as that focal vision task became more difficult. This suggests that some attentional resources are shared between focal vision and ambient vision. This is consistent with the idea discussed earlier that there is a trade-off between focusing and spreading our attention. As we more strongly focus our attention in central vision for greater detail, we trade-off our ambient awareness of our surroundings (Ringer et al., 2016).

There are further complications in understanding the relationship between attention, looking at things, and being aware of them. Recall that overt attention is where you are looking at, but covert attention is when you are attending to something you are not looking at. The fact that covertly attending to things we are not looking at may help to explain, then, why we can fail to notice (i.e., be unaware of) something that we are actually looking straight at (Memmert, 2006). Similarly, research has shown that when something in the visual environment involuntarily captures a viewer’s attention, and they automatically fixate it, their brain may not consciously register having looked at it at all, as shown by both subjective measures of awareness and by their brain waves (i.e., a lack of the “error-related positivity” event related potential) (Belopolsky et al., 2008). Thus, even when a person fixates something, which by definition uses their central vision, it is not always sufficient for their becoming aware of that thing.

So, what can we say about visual awareness, attention, and central and peripheral vision? Overt attention is defined as where we are fixating, which in turn defines central vision, and attention is often necessary for conscious awareness of objects. Thus, attention, awareness, and central vision go hand in hand. Conversely, peripheral vision is often ignored, and thus we are often not consciously aware of the contents of our peripheral visual field, though we use it to navigate through the world. Thus, the focal mode of vision is associated with conscious perception of things in central vision, whereas the ambient mode of vision depends largely on peripheral vision, often without attention, and is largely preconscious.

Nevertheless, the above summary turns out to be somewhat oversimplified. Fixating an object does not guarantee that you are currently attending to it, or consciously aware of it. This is in part because we can expand our attention and awareness into our peripheral visual field. Furthermore, failing to attend to our peripheral visual field can even degrade ambient vision functions such as navigation.

All of these relationships shape our visual experience and awareness of architecture, which is largely determined by the nature and functions of the visual system; an architecture we will suggest is primarily experienced ambiently through our peripheral vision.

## Dividing the House of Architecture

**FIGURE 7:**
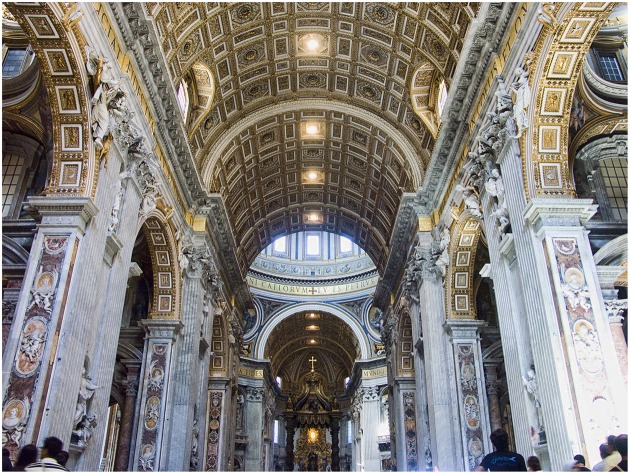
**The Interior of Saint Peters’ Basilica in Rome**. Maderno’s nave, looking toward the chancel. Photo by Jean-Christophe Benoist. https://en.wikipedia.org/wiki/St._Peter’s_Basilica#/media/File:Vatican-StPierre-Intérieur1.jpg.

Our review of visual processing, attention and awareness is intended to divide the consideration of architecture into what we experience through our focal attention to built environments versus our ambient awareness of them. For example, when looking at an image of Saint Peter’s Basilica in Rome, it is possible to come to some judgment about the appearance of the architecture; see **Figure 7**. However, when we are placed inside the space of Saint Peter’s, our surrounding visual environment, processed ambiently, will likely impact our posture, the way we walk through the environment, and our mood, whether consciously or not, in ways that simply looking at a photograph could not achieve. The difference between the above two examples is that the image of Saint Peter’s Basilica relies on our conscious attention as we scan the photograph with our central vision in attempts to find something in the architecture. Conversely, the example of being surrounded in the space relies on using the ambient mode to process our environment. This simple exercise reveals our argument that with architecture there are two distinctly different sets of visual processes which can occur; one through our focal attention which is often prompted by an object-based search for and evaluation of something, and one that ambiently processes the atmosphere of the space which surrounds us. Unfortunately, a lack of consideration of ambient processing of architecture has caused a great deal of trouble in our contemporary understanding of architecture. This is because we have become all too accustomed to regarding architecture only in terms of its focal features with less regard to its atmosphere perceived through ambient processing of its composition of space.

**FIGURE 8:**
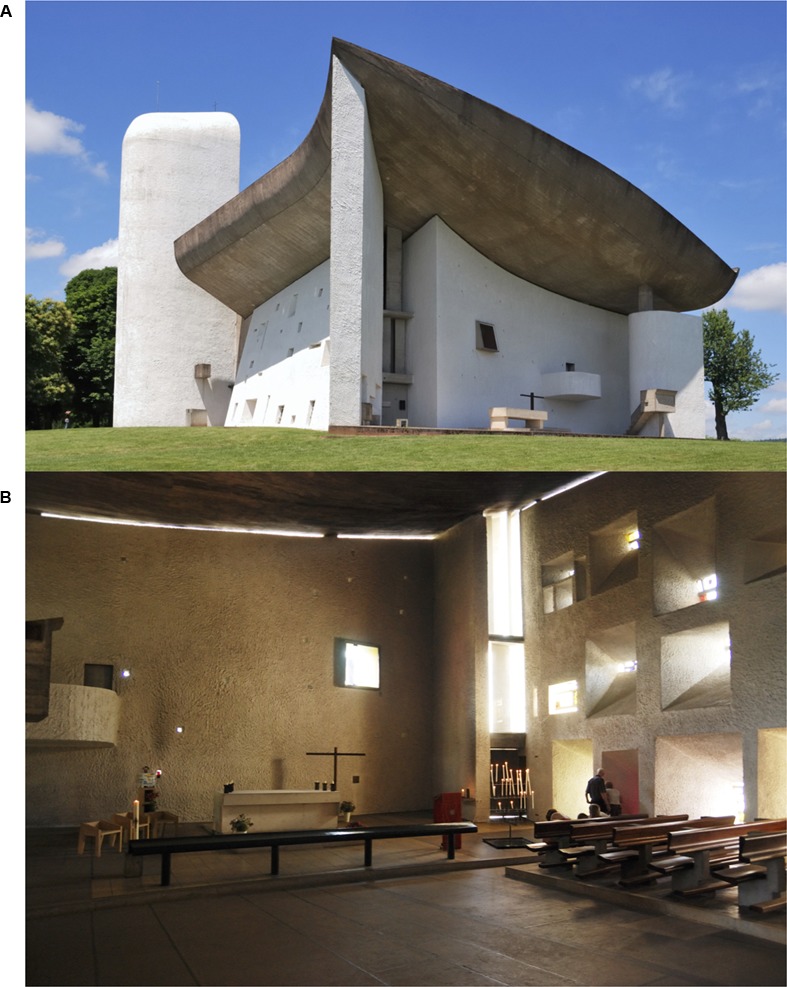
**Le Corbusier’s Chapel of Notre Dame du Haut in Ronchamp**. Exterior view **(A)** and interior view **(B)**. The Chapel can be seen as both object foveated from a distance and spatial atmosphere ambiently processed. Exterior image by Wladyslaw: https://upload.wikimedia.org/wikipedia/de/2/29/Notre_Dame_du_Haut(ws).jpg. Interior image by Wladyslaw: https://de.wikipedia.org/wiki/Datei:Notre_Dame_du_Haut_Innenraum_mit_Altar(ws).jpg.

Having considered research on object and scene perception and focal and ambient processing, what makes architecture unique among the vast array of visual stimuli? The first argument for architecture’s uniqueness is that it can be experienced by all of the above-described forms of visual processing and attention unlike smaller objects like cups. As shown in **Figure 8**, we see a building in the distance as an object, but enter it as a scene for human endeavors. We cast our focal attention on to details of the building when asked our opinion of the architecture, only later to find our ambient awareness consumed by its interior space. The second argument for the uniqueness of architecture is that it is created by humans, and thus architectural objects and spaces are imbued with intention by our perceptual and cognitive systems, unlike beach scenes which are generally absent of human intentions.

The first argument for architecture’s uniqueness brings to light a fascinating problem in the perception and experience of architecture. Our visual system cannot process and become aware of architecture at both levels at once, but it can only do so over time and through exploration (Böhme, 2006; Sauchelli, 2012). As discussed previously regarding focusing attention versus zooming attention out, we trade off visual awareness of the built environment depending on our physical relationship to it (approaching it, versus inhabiting it), and based on how we choose to attend to it (focusing on a detail, or zooming out to perceive it as a space). Interestingly, under this claim, architecture engenders at least two different forms of awareness; one that is intellectual (e.g., focally applying our attention to particular aspects of the architecture) and one that is atmospheric (e.g., ambiently processing the overall mood of the space, either consciously or pre-consciously).

### Focal Attention and Intellectual Analysis of Architecture

**FIGURE 9:**
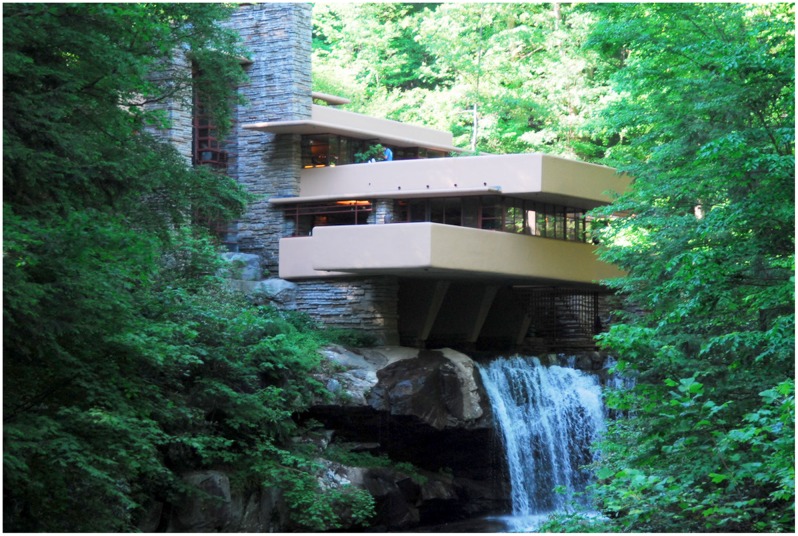
**Frank Lloyd Wright’s Falling Water**. Photo by Somach; https://commons.wikimedia.org/wiki/File:Fallingwater_-_by_Frank_Lloyd_Wright.jpg.

**FIGURE 10:**
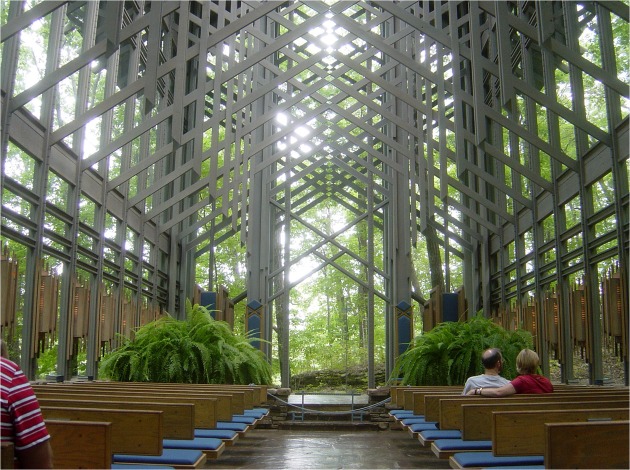
**Interior of Fay Jone’s Thorncrown Chapel in Eureka Springs, Arkansas**. Photo taken by Bobak Ha’Eri: https://en.wikipedia.org/wiki/Thorncrown_Chapel#/media/File:09-02-06-ThorncrownChapel1.jpg.

The intellectual form of focal attention of architecture is probably the most common way people think about architecture when they consider it. When we flip through a book about great architecture, or see a documentary about the works of Frank Lloyd Wright (e.g., see **Figure 9**), or maybe we even happen inside a space such as the Thorncrown Chapel by Fay Jones, we may find ourselves curious about the work (see **Figure 10**). When we think of architecture, we may find ourselves asking basic questions like; *what makes it great*, *how did the designer achieve this*, or even *why does anyone like this building*? This line of questioning prompts a conscious, intellectual attention of the building in which our line of questioning is directed toward an object. This type of engagement is what aesthetic philosopher Roger Scruton (1979) claims as an intellectual form of judgment about a work of architecture. It is intellectual because it is based on our conscious analysis of built features and the particular type of pleasure we find in them. We argue that Scruton’s description of the intellectual process of architectural engagement is best supported by the mechanisms of central vision, the *what pathway*, and focal attention.

Scruton’s construct of *intellect* can be connected to specific widely studied cognitive functions, such as problem solving (e.g., trying to answer the question, “What makes this building great?”), and judgment and decision making (e.g., making a judgment of what is aesthetically pleasing), which involve both logical and heuristic thinking. Thus, Scruton’s argument may create a bridge between what aesthetic philosophers claim about architecture and what psychological scientists refer to as higher order cognition. Note that although the higher-order cognitive processes involved, namely problem solving and judgment and decision making are certainly not considered part of visual perception, the argument is that they are subserved by central vision. This would be similar to the way in which reading comprehension is not a visual process, but the act of reading involves the use of central vision (i.e., reading is generally only possible by using the fovea to fixate each content word in a text–reading is not something people generally do in peripheral vision).

At the very first level of such intellectually driven attention to architecture, we argue that it is very common to direct our attention *to* a built environment using our central visual field when attempting to judge it. For example, trying to answer the question, *what makes this building great*, typically prompts our looking around at it to find the answer, rather like looking for Waldo in a “Where’s Waldo?” image. In doing so, we are primarily utilizing our central visual field and rely heavily on the *what* pathway to distinguish architectural forms and their intersections. In such an intellectual analysis of architecture, the details of the building then come to the forefront of our attention, and this inhibits our overall awareness of the space in favor of what we are looking at with our central vision.

It is possible, of course, that such intellectual processes as Scruton describes may involve peripheral vision, though we think this is unlikely. For example, Scruton argues that the columns at Palazzo Massimi alle Colonne can be perceived as having various rhythms, which can be aesthetically and intellectually appreciated as having architectural value (1979). If the idea that such intellectual engagement with architecture primarily involves central vision is correct, then the aesthetic experience of judging the various rhythms of the same columns should involve making numerous saccades and eye fixations between the columns—a testable hypothesis.

This type of object-based attention of architecture not only occurs when we analyze the great works of architecture, but also extends into everyday life as when we walk into a friend’s house for the first time. We may scan their home in hopes of finding some new piece of information about who they are as if the floor mat at the front door provides greater information about the quality of our friend’s hospitality. Similarly, when we are in the physician’s waiting room, we may look around the space to understand how we will be treated. Environments matter to us, and by scanning them with our central visual field, we allow ourselves to analyze their identity more closely in the same way we might analyze the cup to see if it is full of coffee or tea. The problem with solely relying on this object-based, intellectual attention to our built environment is that it does not tell the complete story of the architecture as it might with the cup of coffee, and in doing so we have come too quick in giving judgment to features which are merely supportive of the larger ambient affect architecture can have on us. The cup of coffee can be known best by our focal attention of it, but the essence of architecture must be found in our full exploration of it as an environment, and it is here where we can distinguish the uniqueness of built environments from other objects, such as the cup of coffee, by the way in which we visually consume them.

**FIGURE 11:**
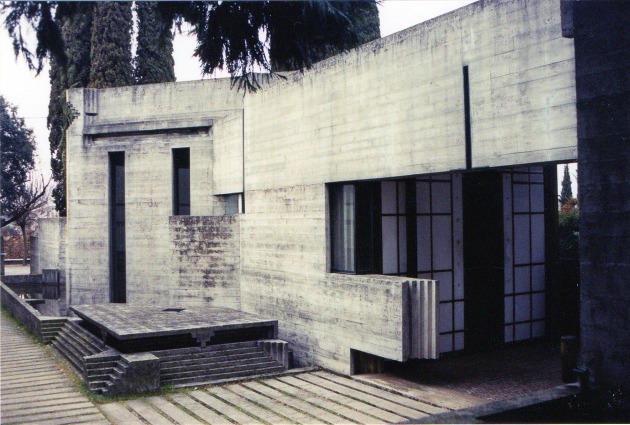
**Exterior of Carlo Scarpa’s Brion Cemetery in San Vito d’Altivole, Italy**. The intense working of ziggurat details are present throughout the Brion Cemetery as seen within the entryway and along the wall and ground. The effect often leads the eye in a chasing game of quick saccades. Photo courtesy of Sandra Rooney.

To illustrate these ideas, consider **Figure 11**, which shows Carlo Scarpa’s Brion Cemetery in San Vito d’Altivole, Italy. If we consider it aesthetically, we find that the details of the design present a highly repetitive ziggurat pattern. The pattern runs through many of the spaces and provides the visual features used by Biederman’s RBC model for object recognition, namely edges and vertices. Such edges, vertices, and corners seem likely to capture our focal attention and eye fixations (Krieger et al., 2000; Mital et al., 2011). If we are judging this built environment while trying to answer the question, “*What makes this building great?”* we may find ourselves drawn into an endless play of edges, captivating our intellectual attention. However, doing so could mislead us into thinking that such details are the foundation of its aesthetic essence. In our assessment, Scarpa, knowingly or not, overloads our attention with the ever presence of edges and vertices. An interesting question is whether we can gain a better feeling of the architectural periphery by exposing our peripheral vision, and thus ambient processing while following the ziggurat pattern. This may in fact be possible, given what has been shown about people’s ability to use their ambient processing to maintain their lane position while driving, even when they are fixating, and focusing their attention on, a control panel below their windshield (Summala et al., 1996). If so, then the ziggurat pattern, by guiding attention and eye movements, may indirectly benefit ambient processing of the entire architectural space. In this sense, the ziggurats might dynamically operate similarly to Jan van Goyen’s placement of the blackbird near the lower center of his painting, as shown in **Figure 4** – namely, the blackbird serves as an ideal viewing position to sense the space of the painting. An alternative possibility, however, is that by increasing the number of foveal details to process in an architectural environment, one decreases the viewer’s attention to the spatial environment as a whole, thus lessening their awareness of it (e.g., Ringer et al., 2016). These alternative hypotheses may not be mutually exclusive if ambient processing occurs without even broadly spread attention in one’s visual periphery (as suggested by the results of Summala et al., 1996). Nevertheless, it would be interesting to empirically test these hypotheses, by measuring attention and ambient processing of an architectural space as a function of the number of interesting details in it available to capture one’s focal attention. Note that regardless of the outcome of such investigations, the current theoretical framework provides theoretical value by asking new and important questions for architecture.

### Ambient Awareness and the Atmosphere of Architecture

The ambient form of awareness with architecture is probably the least common way people consider architecture, and often people must be trained to become consciously aware of the features that they typically process only pre-consciously. In regards to architecture, ambient processing through peripheral vision is probably the vast majority of the way in which we experience built environments in our everyday lives. Going back to Horrey and Wickens (2004) analysis of ambient vision and environmental cues, we can draw an analogy between driving a car and walking through a building in order to analyze the separate information that focal and ambient modes depend on. When it comes to naturally experiencing architecture, it seems likely that many visual tasks involved with built environments do not require direct focal attention, although there are some important exceptions such as keeping track of one’s goal as one navigates through a built environment; opening doors and windows. Often, though, focal attention is directed toward other goals within the built environment, such as speaking with people (e.g., attention to facial expressions), object interaction (e.g., reaching for a cup to drink), or even introspection (e.g., thinking/daydreaming). Returning to Scruton’s (1979) argument that the appreciation of architecture involves an intellectual process, this suggests that we must inspect architecture with the focal mode of vision to assess its quality. However, a more naturalistic experience of architecture through the ambient mode suggests a non-intellectual analysis because the ambient mode does not require focal attention, and likely operates pre-consciously (Previc, 1998).

Previc’s (1998) model of space perception includes ambient processing for maintaining spatial orientation and postural control, and describes the extension of our body into our environment. It relies on stability of the ambient environment as predominately perceived through peripheral vision and preconsciously. The Pantheon in Rome is a good example as the lack of vertical cues and the visual tendency to look up toward the oculus in the ceiling reduces our ambient perception of spatial orientation and postural control (e.g., Leibowitz and Post, 1982) destabilizing our posture which may cause us to become more aware of the space. Under Previc’s model, our body movement adjusts our perception of the world and the built environment, processed ambiently, adjusts our body movement.

In the above descriptions, ambient awareness of architecture refers both to the ambient processes discussed by Horrey and Wickens (2004) and a particular range of aesthetic feelings we have regarding an architectural environment, which we are occasionally made aware of when experiencing architecture as an atmosphere.

A good example of such architectural atmosphere is provided by consideration of Fay Jones’ Thorncrown Chapel in Eureka Springs, Arkansas (**Figure 10**). In looking at it, we may find ourselves intrigued by the detail of joinery and the negative intersections at the trusses, but we argue that this intellectual, object-based attention is not the reason for the ambient feeling of atmosphere we might gain by being in the space. The quality of light, the height and width of the space, the movement of trees seen peripherally through the expanse of windows, the rhythmic pattern of frames cascading forward all aid in producing the essential feeling we gain from the atmosphere in the chapel. Only through these features, accessible through the peripheral visual field, processed by the *where* pathway, and with ambient vision, do we fully experience the atmosphere of the space. Our intellectual attention only provides the individual details of evidence that our friend is indeed welcoming us into their home which we experience ambiently as a space. This is much the same way that Fay Jones welcomes us to his own atmosphere of aspired transcendence with his design of the Thorncrown Chapel (Foley, 2010). Fittingly, Fay Jones himself was quoted as asking his daughter “Do you feel it?” as they entered the space together (Foley, 2010).

This raises two critical questions for our theory: (1) What exactly is the *atmosphere* of a built environment, and (2) Is architectural atmosphere amenable to scientific inquiry? As noted by Tröndle and Tschacher (2012), in everyday conversation we commonly talk about the atmosphere of a place using words such as bright, cheerful, dreary, or gloomy, but *atmosphere* is a relatively new theoretical construct in the aesthetics of architecture (Böhme, 2006) and geography (Anderson, 2009) with philosophical consideration dating back to August Schmarsow’s idea of spatial essence (Schmarsow, 1873). Böhme (2006, p. 402) defines atmosphere as “the character of the space in which we find ourselves” and “an affective tendency by which our mood is attuned to the nature of a space...” In simpler terms “by entering a room, one may adopt a certain mood” (Tröndle and Tschacher, 2012, p. 106). Furthermore, the atmosphere of a space is influenced not only by the structure of the built environment (i.e., the bricks and mortar), but also by the light in it, which “…give[s] a space a distinctive character. Light that fills a room can make that room serene, exhilarating, gloomy, festive, or eerie” (Böhme, 2006, p. 405). Thus, atmosphere, as discussed both in the aesthetics of architecture, and in everyday conversation, is the overall emotional impression, or mood, a person feels in a built environment. Importantly, recent work by Tröndle and Tschacher (2012) shows promising results in terms of empirically measuring the effects of manipulating the atmosphere of a room.

For much of the 20th century, most experimental psychologists considered emotion to be outside of the realm of scientific investigation, on the assumption that it could only be approached qualitatively or intuitively. However, over the last several decades it has become abundantly clear that emotions are amenable to scientific investigation and explanation. Importantly, specific emotions (e.g., disgust, fear, happiness, sadness, anger, surprise) can be reliably evoked in different people by particular stimuli such as film clips (Hewig et al., 2005; Coan and Allen, 2007). Just as importantly, those emotions can be measured reliably with both subjective measures (e.g., ratings scales) and objective measures (e.g., facial expressions, heart rate, galvanic skin response, EEG, or fMRI) (Dimberg et al., 2000; Coan and Allen, 2007; Lee and Hsieh, 2014; Saarimäki et al., 2016). Concerning atmosphere, moods are distinguishable from emotions by being longer lasting (e.g., from several minutes to several hours), being less intensely felt (often experienced as a background feeling), and by having causes that are not always easily identifiable by the person feeling them (Mitchell and Phillips, 2007). Finally, a large body of research has shown that emotions (and moods) have distinct dimensions of valence (i.e., positive to negative emotions) and arousal (low to high), and the more recently proposed dimension of dominance (low to high) (Koelstra et al., 2012). For example, a joyful mood would have high positive valence, high arousal, and high dominance. Conversely, a subdued dark mood would have a moderately negative valence, relatively low arousal, and low dominance. Thus, to the extent that architectural environments affect people’s moods, that should constitute the environment’s atmosphere, and should be amenable to scientific study.

We propose that the atmosphere, or mood, of a built environment is particularly processed by ambient vision from the visual periphery. As noted above, aestheticians (e.g., Böhme, 2006; Anderson, 2009) have argued that an architectural atmosphere is the mood evoked by perception of its space. Concerning space perception, we have reviewed evidence showing that peripheral vision is important for holistic processing of the “gist” of scenes (Larson and Loschky, 2009), identifying the locations of scenes (Eberhardt et al., 2016), and creating an accurate mental representation of a space when walking through it (Fortenbaugh et al., 2007, 2008). We have also reviewed evidence showing how peripheral vision is important for ambient vision functions such as avoiding obstacles while walking, and maintain one’s posture and balance in a space. All of this indicates that ambient processing of peripheral vision is important for holistic processing of the *space* of a built environment.

This leads us to the next question, which is whether ambient processing of a space through peripheral vision allows people to perceive the *mood* of that space, and research suggests that the answer is yes. This conclusion comes from numerous studies that have investigated the processing of emotional scenes in peripheral vision. Typically, such emotional scenes come from the International Affective Picture System (Lang et al., 2008), which includes many photographs of scenes varying in both valence (positive vs. negative) and arousal (low vs. high), for example, a man and a woman drinking coffee (neutral valence, low arousal), a man attacking a woman (negative valence, high arousal), or a man and a woman making love (positive valence, high arousal). These studies have shown that emotional scenes in the visual periphery can involuntarily capture attention and eye movements (Nummenmaa et al., 2010; McSorley and van Reekum, 2013; but see Acunzo and Henderson, 2011), even when viewers are instructed not to look at them (Kissler and Keil, 2008). Furthermore, these effects of emotional scenes presented in peripheral vision can occur even when viewers are unable to report details of the emotional content (e.g., that a man attacked a woman) (Calvo, 2006; Calvo et al., 2008; Rigoulot et al., 2008; McSorley and van Reekum, 2013). Importantly, however, such emotional processing of peripheral vision is degraded when a person’s attentional resources are focused in central vision (Calvo and Lang, 2005), which suggests that peripheral visual processing of emotional content requires some allocation of attention. In sum, while peripheral vision is often insufficient to identify emotionally charged objects or entities in a scene, it does allow processing of a holistic emotional gist of a scene, which can involuntarily draw people’s attention to those emotional stimuli. We therefore hypothesize that the emotional gist of a scene is like the scene’s mood, and thus that peripheral vision can play an important role in perceiving the atmosphere of a built environment.

The above discussion leaves two important unanswered questions for our theory. First, using only peripheral vision, can viewers recognize the mood, or atmosphere of a built environment when it is not determined by specific emotionally charged content (e.g., a man attacking a woman), but instead is determined by architecturally relevant variables (e.g., the architect’s use of light, space, and materials)? We believe that it should be possible to do so, but this is an empirical question needing to be tested. Second, if the answer to the preceding question is “yes,” then is it also possible for viewers to perceive architectural mood, or atmosphere, without paying attention to their peripheral vision? The results of Calvo and Lang (2005) described above suggest the answer is “no,” but work on ambient vision has shown that some activities using purely peripheral vision (e.g., walking while texting, or maintaining one’s lane position while driving and looking at a display below the dashboard) are at least moderately preserved even when viewers strongly focus their attention on the fovea (Summala et al., 1996; Plummer et al., 2015). Thus, our second question also needs to be tested.

## Experimental Design Considerations

It is important that we eventually test the above proposed theoretical distinction between foveated intellectual attention of designed details versus peripheral ambient atmosphere experience of architectural spaces. Here we sketch out two possible ways to test these ideas which could be achieved by recruiting lesion patients that are blinded along the peripheral visual field/dorsal stream or the central visual field/ventral stream, or recruiting visually healthy participants and applying window and scotoma blinding via eye tracking.

Some examples of research regarding lesion patients include optic ataxia patients with dorsal lesions who showed the inability to distinguish locations of objects (Bálint and Harvey, 1995) as well as ventral lesions resulting in visual form agnosia with patients showing the inability to recognize objects (Benson and Greenberg, 1969). Our interest in these studies is focused on the inability of these patients to experience visual stimuli presented in the visual field associated with the blindness caused by the lesion. In the case of optic ataxia, these patients should encounter problems associated with perceiving the composition of architectural spaces and perceiving the mood of the space, given the proposed hypothesis that the composition of architectural spaces is critical to providing an atmosphere that affects the mood of someone viewing it. On the other hand, patients suffering from visual form agnosia should have great difficulty apprehending the intellectual appreciation of details presented foveally, but may still be affected by the surrounding space peripherally via the atmosphere created, compositionally, by the architect. One caveat is that even though a patient has a lesion, neural networks are known to reroute in compensation for lesion damage and may provide partially regained perception, even if unconsciously (O’Regan and Noë, 2001). Thus, follow-up research should also present window / scotoma conditions to both lesion patients and normally sighted viewers.

**FIGURE 12:**
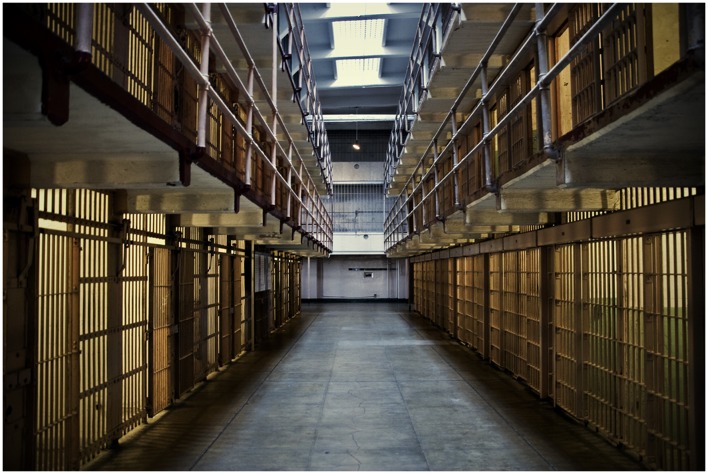
**Interior of Alcatraz Prison, Alcatraz Island, CA, USA**. This built environment evokes an atmosphere describable in almost exclusively negatively valenced terms. Photo taken by Krystian Olszanski (CC BY 2.0). https://www.flickr.com/photos/krystiano/4637560951/in/photostream.

With either lesion patients and/or window/scotoma conditions, we could experimentally test the validity of our proposed distinction by showing viewers architectural images while varying the visual field available to them (e.g., central vision through a window condition and peripheral vision through a scotoma condition) and measuring an array of dependent variables that assess viewers’ intellectual responses (e.g., concerning architectural details and qualities) versus mood-related responses (e.g., arousal and valence). Importantly, the architectural images used should vary in terms of their details (which are best seen foveally) and their spaces (which are best seen peripherally), and in terms of their judged architectural qualities (as determined ahead of time by a group of architectural experts) and their mood (as determined ahead of time by a group of architecturally naïve viewers). For example, the images should contain recognizable architectural “objects” (e.g., details such as column types: ionic vs. corinthian; intersection of materials: wood detail meeting a metal detail) and have reliably identifiable moods (e.g., **Figure 12**: Alcatraz prison: oppressive, gloomy, depressing; **Figure 10**: Thorncrown Chapel: calm, meditative, transcendental).

Then, one could show three groups of viewers the same set of architectural images in the window or scotoma conditions (e.g., a scotoma group, a window group, and a whole image control group). Importantly, the image sets should be derived from panoramic photos and presented on a large screen (e.g., 180° diameter) to fully engage the peripheral visual field during presentation. However, in the window condition, only a circular region in the central 10° diameter (out to 5° eccentricity) of the panoramic images would be shown, with the remainder of the 180° wide image would be replaced with even gray. The scotoma condition would be the inverse of the window condition – the central 10° circular region would be filled with neutral gray, but the remainder of the 180° wide image would be visible.

Images would be briefly flashed for the duration of roughly one eye fixation (e.g., 330 ms), in order to engage only central or peripheral vision. Such methods would be very similar to those in a recent set of experiments by Loschky et al. (2015), though those were not concerned with the perception of architecture, but rather the rapid categorization of scenes. Then, in order to assess intellectual versus mood-related responses, think-aloud protocols would be a good initial source of data. For these, one could simply ask viewers to describe anything that comes to mind from the image they just saw. Transcripts of their think-aloud protocols could then be analyzed in terms of the types of words used, or concepts described. A key question would be whether architectural details and qualities would be more frequently described in the window condition, and whether moods or spatial terms would be more frequently described in the scotoma condition. To assess viewers’ moods as affected by the architectural views, we could also use both subjective emotion rating scales and objective biometric measures (Coan and Allen, 2007). Biometric measures could include galvanic skin response, heart rate variability, and breathing rate variability as measures of arousal, and EMG facial readings (Dimberg et al., 2000) or EEG connectivity as measures of valence (Lee and Hsieh, 2014).

In the window condition, in which only central vision is available, we would expect that think-aloud protocols would include mention of important targeted details within the architectural image, such as the detailed intersection of wooden trusses with the metal bracket at the Thorncrown Chapel designed by Fay Jones. Alternately, in the scotoma condition, which only allows peripheral vision, we would expect that verbal protocols would be more likely to include descriptions of important atmospheric qualities of the architecture, which are readily available through peripheral processing, such as the transcendental mood that the Thorncrown Chapel architectural space was designed to evoke. In terms of subjective ratings of emotions and biometric measures of arousal and valence, we would expect that they would be more influenced by the scotoma condition, based on the use of peripheral vision, than by the window condition, based on the use of central vision. Thus, it will be of great interest to determine whether the control condition, in which viewers see each entire image, shows a discrepancy between the contents of the verbal protocols and the mood measures. Given that central vision tends to be what we pay attention to, thus leading to an intellectual attention of the details that are being foveated, viewers’ verbal protocols might be more similar to the window (central vision) condition, while the mood measures might be more similar to the scotoma (peripheral vision) condition. Another interesting possibility would be to produce hybrid images that combine a) architectural details from one building in the central window region with b) the architectural space from another building shown in the periphery. A key question would be whether the think-aloud protocols and mood measures would be more influenced by the central and peripheral regions, respectively.

Additionally, we suggest that images be grouped into similar mood signatures and presented in blocks over time. For example, calm images would be shown for an extended time period (each at 330 ms for a 10 min session), then tense images (each at 330 ms for another 10 min session). The use of 10 min intervals for each set of images per mood signature is much like Kreibig et al. (2007), who studied cardiovascular, electrodermal, and respiratory response patterns to fear and sadness inducing films. In their study, Kreibig et al. (2007) used 10 min per film to test either fear or sadness.

To study the effects of attention on perception of atmosphere, we could use dual task methods, such as having participants respond to a visual task in central vision, such as on their cell phone, versus simply having participants respond to the architectural images.

Finally, because ambient processing is critically important for navigating in our environment, other studies should investigate the role of peripheral vision in perceiving the mood of an architectural space while navigating through it. This could be done using the window, scotoma, and control conditions in a helmet-based virtual reality, and with the same array of subjective and objective measures of ambient mood and focal intellectual attention.

## Conclusion

In summary, most of the time we attend to and are aware of the things we are looking at in central vision. This applies to architecture as well and often occurs when we look at architecture to make some type of intellectual judgment about its appearance. Conversely, in our everyday experience of architecture, we usually do not pay attention to it – architecture is usually treated as background. Nevertheless, the background space, processed by our peripheral vision in some sort of pre-conscious state (Previc, 1998), may affect our mood (e.g., we may feel closed in or depressed because the building appears dreary). However, in some cases of outstanding architecture, our attention will be grabbed by the entirety of a built space, such as the interior of the Pantheon. In those cases, our attention can expand outward from central vision to encompass all or most of our visual field, which is largely in peripheral vision. At such moments, we may become aware of the space we inhabit, achieving a conscious ambient awareness of the atmosphere created by the architecture.

Given the mechanisms of vision from the retina to the dorsal and ventral streams, the experience of architecture simultaneously operates within the central and peripheral visual fields through focal and ambient modes of vision. Due to this fluid state, architecture stands as a unique type of environmental stimulus which can transition between object and scene perception along a cognitive spectrum of visual and attentive responses unlike most other stimuli. Our specific claim is that there is an architecture which we intellectually assess through our focal attention of whatever we look at (foveate) and an architecture which we are affected by through the atmosphere felt through ambient processing of our surrounding environment. Importantly, these separate forms of architectural processing operate in parallel throughout the visual perceptual system. Thus, a complete understanding of architecture must consider it as a stimulus whose perception is dependent on the relationship between our focal and ambient processing, each of which plays a unique role in our visual experience of architecture. Our aim is to balance the understanding of architectural experience by bringing attention to the essential ambient processing of peripheral vision, which is often more difficult to describe and often neglected, but which we believe has a profound effect on our experience of built environments.

## Author Contributions

Developed the theory and hypotheses: KR, LL. Wrote the paper: KR, LL. Discussed ideas in the paper: KR, LL, RC. Proof-read the paper: KR, LL, RC.

## Conflict of Interest Statement

The authors declare that the research was conducted in the absence of any commercial or financial relationships that could be construed as a potential conflict of interest.

## References

[B1] Acunzo D. J. Henderson J. M. (2011). No emotional “pop-out” effect in natural scene viewing. *Emotion* 11 1134–1143. 10.1037/a0022586 21787079

[B2] Anderson B. (2009). Affective atmospheres. *Emot. Space Soc.* 2 77–81. 10.1016/j.emospa.2009.08.005

[B3] Arcaro M. J. McMains S. A. Singer B. D. Kastner S. (2009). Retinotopic organization of human ventral visual cortex. *J. Neurosci.* 29 10638–10652. 10.1523/JNEUROSCI.2807-09.2009 19710316 PMC2775458

[B4] Baldassano C. Fei-Fei L. Beck D. M. (2016). Pinpointing the peripheral bias in neural scene-processing networks during natural viewing. *J. Vis.* 16:9 10.1167/16.2.9 27187606

[B5] Bálint R. Harvey M. (1995). Psychic paralysis of gaze, optic ataxia, and spatial disorder of attention. *Cogn. Neuropsychol.* 12 265–281. 10.1080/02643299508251999

[B6] Bánréti-Fuchs K. (1967). Perception without awareness. *Acta Psychol.* 26 148–160. 10.1016/0001-6918(67)90013-3 6037307

[B7] Barton K. R. Valtchanov D. Ellard C. (2014). Seeing beyond your visual field: the influence of spatial topology and visual field on navigation performance. *Environ. Behav.* 46 507–529. 10.1177/0013916512466094

[B8] Belopolsky A. V. Kramer A. F. Theeuwes J. (2008). The role of awareness in processing of oculomotor capture: evidence from event-related potentials. *J. Cogn. Neurosci.* 20 2285–2297. 10.1162/jocn.2008.20161 18457508

[B9] Benson D. F. Greenberg J. P. (1969). Visual form agnosia: a specific defect in visual discrimination. *Arch. Neurol.* 20 82–89. 10.1001/archneur.1969.00480070092010 4303441

[B10] Biederman I. (1987). Recognition-by-components: a theory of human image understanding. *Psychol. Rev.* 94 115–147. 10.1037/0033-295X.94.2.115 3575582

[B11] Böhme G. (2006). “Atmosphere as the subject matter of architecture,” in *Herzog & DeMeuron: Natural History*, ed. Ursprung P. (Zurich: Lars Müller Publishers), 398–407.

[B12] Boucart M. Lenoble Q. Quettelart J. Szaffarczyk S. Despretz P. Thorpe S. J. (2016). Finding faces, animals, and vehicles in far peripheral vision. *J. Vis.* 16:10 10.1167/16.2.10 27404483

[B13] Bouma H. (1970). Interaction effects in parafoveal letter recognition. *Nature* 226 177–178. 10.1038/226177a0 5437004

[B14] Calvo M. G. (2006). Processing of emotional visual scenes outside the focus of spatial attention: the role of eccentricity. *Vis. Cogn.* 13 666–676. 10.1080/13506280500418413

[B15] Calvo M. G. Lang P. J. (2005). Parafoveal semantic processing of emotional visual scenes. *J. Exp. Psychol.* 31 502–519. 10.1037/0096-1523.31.3.502 15982128

[B16] Calvo M. G. Nummenmaa L. Hyönä J. (2008). Emotional scenes in peripheral vision: selective orienting and gist processing, but not content identification. *Emotion* 8 68–80. 10.1037/1528-3542.8.1.68 18266517

[B17] Carrasco M. (2011). Visual attention: the past 25 years. *Vis. Res.* 51 1484–1525. 10.1016/j.visres.2011.04.012 21549742 PMC3390154

[B18] Chun M. M. Golomb J. D. Turk-Browne N. B. (2011). A taxonomy of external and internal attention. *Annu. Rev. Psychol.* 62 73–101. 10.1146/annurev.psych.093008.100427 19575619

[B19] Coan J. A. Allen J. J. (2007). *Handbook of Emotion Elicitation and Assessment.* Oxford: Oxford university press.

[B20] Coy A. Ringer R. V. Larson A. M. Luczak M. Loschky L. C. (2014). Investigating visual crowding of objects in complex scene images [Abstract]. *J. Vis.* 14 779–779. 10.1167/14.10.779

[B21] Curcio C. A. Sloan K. R. Kalina R. E. Hendrickson A. E. (1990). Human photoreceptor topography. *J. Comp. Neurol.* 292 497–523. 10.1002/cne.902920402 2324310

[B22] Dimberg U. Thunberg M. Elmehed K. (2000). Unconscious facial reactions to emotional facial expressions. *Psychol. Sci.* 11 86–89. 10.1111/1467-9280.00221 11228851

[B23] Drasdo N. Fowler C. W. (1974). Non-linear projection of the retinal image in a wide-angle schematic eye. *Br. J. Ophthalmol.* 58 709–714. 10.1136/bjo.58.8.709 4433482 PMC1215006

[B24] Eberhardt S. Zetzsche C. Schill K. (2016). Peripheral pooling is tuned to the localization task. *J. Vis.* 16 14–14. 10.1167/16.2.14 27902838

[B25] Ehinger K. A. Rosenholtz R. (2016). A general account of peripheral encoding also predicts scene perception performance. *J. Vis.* 16:13 10.1167/16.2.13 27893077

[B26] Eriksen C. W. Yeh Y. Y. (1985). Allocation of attention in the visual field. *J. Exp. Psychol.* 11 583–597. 10.1037/0096-1523.11.5.583 2932532

[B27] Foley L. (2010). *Sacred Spaces: The Architecture of Fay Jones.* Fayetteville, AR: University of Arkansas Press.

[B28] Fortenbaugh F. C. Hicks J. C. Hao L. Turano K. A. (2007). Losing sight of the bigger picture: peripheral field loss compresses representations of space. *Vision Res.* 47 2506–2520. 10.1016/j.visres.2007.06.012 17692884 PMC2693205

[B29] Fortenbaugh F. C. Hicks J. C. Turano K. A. (2008). The effect of peripheral visual field loss on representations of space: evidence for distortion and adaptation. *Invest. Ophthalmol. Vis. Sci.* 49 2765–2772. 10.1167/iovs.07-1021 18515599

[B30] Franchak J. M. Adolph K. E. (2010). Visually guided navigation: head-mounted eye-tracking of natural locomotion in children and adults. *Vis. Res.* 50 2766–2774. 10.1016/j.visres.2010.09.024 20932993 PMC3013502

[B31] Frisby J. P. Stone J. V. (2010). *Seeing: The Computational Approach to Biological Vision.* Cambridge, MA: The MIT Press.

[B32] Geringswald F. Porracin E. Pollmann S. (2016). Impairment of visual memory for objects in natural scenes by simulated central scotomata. *J. Vis.* 16:6 10.1167/16.2.6 27002551

[B33] Goodale M. A. Milner A. D. (1992). Separate visual pathways for perception and action. *Trends Neurosci.* 15 20–25. 10.1016/0166-2236(92)90344-8 1374953

[B34] Grill-Spector K. Kourtzi Z. Kanwisher N. (2001). The lateral occipital complex and its role in object recognition. *Vis. Res.* 41 1409–1422. 10.1016/S0042-6989(01)00073-6 11322983

[B35] Hewig J. Hagemann D. Seifert J. Gollwitzer M. Naumann E. Bartussek D. (2005). A revised film set for the induction of basic emotions. *Cogn. Emot.* 19 1095–1109. 10.1080/02699930541000084

[B36] Horrey W. J. Wickens C. D. (2004). Focal and ambient visual contributions and driver visual scanning in lane keeping and hazard detection. *Proc. Hum. Fact. Ergon. Soc. Annu. Meet.* 48 2325–2329. 10.1177/154193120404801926

[B37] Horrey W. J. Wickens C. D. Consalus K. P. (2006). Modeling drivers’ visual attention allocation while interacting with in-vehicle technologies. *J. Exp. Psychol.* 12 67–78. 10.1037/1076-898x.12.2.67 16802889

[B38] Ikeda M. Takeuchi T. (1975). Influence of foveal load on the functional visual field. *Percept. Psychophys.* 18 255–260. 10.3758/BF03199371

[B39] Kissler J. Keil A. (2008). Look-don’t look! How emotional pictures affect pro- and anti-saccades. *Exp. Brain Res.* 188 215–222. 10.1007/s00221-008-1358-0 18368396

[B40] Koelstra S. Mühl C. Soleymani M. Lee J. S. Yazdani A. Ebrahimi T. (2012). Deap: a database for emotion analysis; using physiological signals. *IEEE Trans. Affect. Comput.* 3 18–31. 10.1109/T-AFFC.2011.15

[B41] Kreibig S. D. Wilhelm F. H. Roth W. T. Gross J. J. (2007). Cardiovascular, electrodermal, and respiratory response patterns to fear-and sadness-inducing films. *Psychophysiology* 44 787–806. 10.1111/j.1469-8986.2007.00550.x 17598878

[B42] Krieger G. Rentschler I. Hauske G. Schill K. Zetzsche C. (2000). Object and scene analysis by saccadic eye-movements: an investigation with higher-order statistics. *Spat. Vis.* 13 201–214. 10.1163/156856800741216 11198232

[B43] Kuhn G. Findlay J. M. (2009). Misdirection, attention and awareness: inattentional blindness reveals temporal relationship between eye movements and visual awareness. *Q. J. Exp. Psychol.* 63 136–146. 10.1080/17470210902846757 19459083

[B44] Lamble D. Kauranen T. Laakso M. Summala H. (1999). Cognitive load and detection thresholds in car following situations: safety implications for using mobile (cellular) telephones while driving. *Accid. Anal. Prev.* 31 617–623. 10.1016/S0001-4575(99)00018-4 10487336

[B45] Lang P. J. Bradley M. M. Cuthbert B. N. (2008). *International Affective Picture System (IAPS): Affective Ratings of Pictures and Instruction Manual.* Technical Report A-8 University of Florida, Gainesville, FL.

[B46] Larson A. M. Freeman T. E. Ringer R. V. Loschky L. C. (2014). The spatiotemporal dynamics of scene gist recognition. *J. Exp. Psychol.* 40 471–487. 10.1037/a0034986 24245502

[B47] Larson A. M. Loschky L. C. (2009). The contributions of central versus peripheral vision to scene gist recognition. *J. Vis.* 9:6 10.1167/9.10.6 19810787

[B48] Larsson J. Heeger D. J. (2006). Two retinotopic visual areas in human lateral occipital cortex. *J. Neurosci.* 26 13128–13142. 10.1523/JNEUROSCI.1657-06.2006 17182764 PMC1904390

[B49] Lee Y. Y. Hsieh S. (2014). Classifying different emotional states by means of EEG-based functional connectivity patterns. *PLoS ONE* 9:e95415 10.1371/journal.pone.0095415 PMC3990628 24743695

[B50] Leibowitz H. W. Post R. B. (1982). “The two modes of processing concept and some implications,” in *Organization and Representation in Perception*, ed. Beck J. (Hillsdale, NJ: Erlbaum), 343–363.

[B51] Livingstone M. Hubel D. (1988). Segregation of form, color, movement, and depth: anatomy, physiology, and perception. *Science* 240 740–749. 10.1126/science.3283936 3283936

[B52] Loschky L. C. Boucart M. Szaffarczyk S. Beugnet C. Johnson A. Tang J. L. (2015). The contributions of central and peripheral vision to scene gist recognition with a 180° visual field [Abstract]. *J. Vis.* 15:570 10.1167/15.12.570 31100131

[B53] Loschky L. C. Nuthmann A. Fortenbaugh F. C. Levi D. M. (2017). Scene perception from central to peripheral vision. *J. Vis.* 17:6 10.1167/17.1.6 28114488

[B54] Malach R. Levy I. Hasson U. (2002). The topography of high-order human object areas. *Trends Cogn. Sci.* 6 176–184. 10.1016/S1364-6613(02)01870-3 11912041

[B55] Marigold D. S. Weerdesteyn V. Patla A. E. Duysens J. (2007). Keep looking ahead? Re-direction of visual fixation does not always occur during an unpredictable obstacle avoidance task. *Exp. Brain Res.* 176 32–42. 10.1007/s00221-006-0598-0 16819646

[B56] McSorley E. van Reekum C. M. (2013). The time course of implicit affective picture processing: an eye movement study. *Emotion* 13 769–773. 10.1037/a0032185 23527504

[B57] Memmert D. (2006). The effects of eye movements, age, and expertise on inattentional blindness. *Conscious. Cogn.* 15 620–627. 10.1016/j.concog.2006.01.001 16487725

[B58] Merikle P. M. Smilek D. Eastwood J. D. (2001). Perception without awareness: perspectives from cognitive psychology. *Cognition* 79 115–134. 10.1016/S0010-0277(00)00126-8 11164025

[B59] Mishkin M. Ungerleider L. G. Macko K. A. (1983). Object vision and spatial vision: two cortical pathways. *Trends Neurosci.* 6 414–417. 10.1016/0166-2236(83)90190-X

[B60] Mital P. Smith T. J. Hill R. Henderson J. M. (2011). Clustering of gaze during dynamic scene viewing is predicted by motion. *Cogn. Comput.* 3 5–24. 10.1167/13.8.16

[B61] Mitchell R. L. C. Phillips L. H. (2007). The psychological, neurochemical and functional neuroanatomical mediators of the effects of positive and negative mood on executive functions. *Neuropsychologia* 45 617–629. 10.1016/j.neuropsychologia.2006.06.030 16962146

[B62] Nasr S. Liu N. Devaney K. J. Yue X. Rajimehr R. Ungerleider L. G. (2011). Scene-selective cortical regions in human and nonhuman primates. *J. Neurosci.* 31 13771–13785. 10.1523/jneurosci.2792-11.2011 21957240 PMC3489186

[B63] Nummenmaa L. Hyönä J. Calvo M. G. (2010). Semantic categorization precedes affective evaluation of visual scenes. *J. Exp. Psychol. Gen.* 139 222–246. 10.1037/a0018858 20438250

[B64] O’Regan J. K. Noë A. (2001). A sensorimotor account of vision and visual consciousness. *Behav. Brain Sci.* 24 939–973. 10.1017/S0140525X01000115 12239892

[B65] Palmer S. E. (1999). *Vision Science: Photons to Phenomenology.* Cambridge, MA: MIT Press.

[B66] Pashler H. E. (1998). *The Psychology of Attention.* Cambridge, MA: The MIT Press.

[B67] Pelli D. G. Tillman K. A. (2008). The uncrowded window of object recognition. *Nat. Neurosci.* 11 1129–1135. 10.1038/nn.2187 18828191 PMC2772078

[B68] Pitzalis S. Sereno M. I. Committeri G. Fattori P. Galati G. Patria F. (2010). Human V6: the medial motion area. *Cereb. Cortex* 20 411–424. 10.1093/cercor/bhp112 19502476 PMC2803738

[B69] Pitzalis S. Sereno M. I. Committeri G. Fattori P. Galati G. Tosoni A. (2013). The human homologue of macaque area V6A. *Neuroimage* 82 517–530. 10.1016/j.neuroimage.2013.06.026 23770406 PMC3760586

[B70] Plummer P. Apple S. Dowd C. Keith E. (2015). Texting and walking: effect of environmental setting and task prioritization on dual-task interference in healthy young adults. *Gait & Posture* 41 46–51. 10.1016/j.gaitpost.2014.08.007 25193796

[B71] Posner M. I. (1980). Orienting of attention. *Q. J. Exp. Psychol.* 32 3–25. 10.1080/00335558008248231 7367577

[B72] Previc F. H. (1998). The neuropsychology of 3-D space. *Psychol. Bull.* 124 123–164. 10.1037/0033-2909.124.2.123 9747184

[B73] Rigoulot S. Delplanque S. Despretz P. Defoort-Dhellemmes S. Honoré J. Sequeira H. (2008). Peripherally presented emotional scenes: a spatiotemporal analysis of early ERP responses. *Brain Topogr.* 20 216–223. 10.1007/s10548-008-0050-9 18335307

[B74] Ringer R. V. Throneburg Z. Johnson A. P. Kramer A. F. Loschky L. C. (2016). Impairing the useful field of view in natural scenes: tunnel vision versus general interference. *J. Vis.* 16:7 10.1167/16.2.7 27050950

[B75] Saarimäki H. Gotsopoulos A. Jääskeläinen I. P. Lampinen J. Vuilleumier P. Hari R. (2016). Discrete neural signatures of basic emotions. *Cereb. Cortex* 26 2563–2573. 10.1093/cercor/bhv086 25924952

[B76] Sauchelli A. (2012). The structure and content of architectural experience: scruton on architecture as art. *Estetika* 49 26–44.

[B77] Schmarsow A. (1873). “The essence of architectural creation,” in *Empathy, Form and Space–Problems in German Aesthetics 1873–1893*, ed. Mallgrave H. F. (Santa Monica, CA: The Getty Center for the History of Art and the Humanities), 125–148.

[B78] Schyns P. G. Oliva A. (1994). From blobs to boundary edges: evidence for time- and spatial-scale-dependent scene recognition. *Psychol. Sci.* 5 195–200. 10.1111/j.1467-9280.1994.tb00500.x

[B79] Scruton R. (1979). *The Aesthetics of Architecture.* Princeton, NJ: Princeton University Press.

[B80] Siegelbaum S. A. Hudspeth A. J. (2000). *Principles of Neural Science* Vol. 4 eds Kandel E. R. Schwartz J. H. Jessell T. (New York, NY: McGraw-hill), 1227–1246.

[B81] Strasburger H. Rentschler I. Jüttner M. (2011). Peripheral vision and pattern recognition: a review. *J. Vis.* 11:13 10.1167/11.5.13 PMC11073400 22207654

[B82] Summala H. Lamble D. Laakso M. (1998). Driving experience and perception of the lead car’s braking when looking at in-car targets. *Accid. Anal. Prev.* 30 401–407. 10.1016/S0001-4575(98)00005-0 9666236

[B83] Summala H. Nieminen T. Punto M. (1996). Maintaining lane position with peripheral vision during in-vehicle tasks. *Hum. Fact.* 38 442–451. 10.1518/001872096778701944

[B84] Tröndle M. Tschacher W. (2012). The physiology of phenomenology: the effects of artworks. *Empir. Stud. Arts* 30 75–113. 10.2190/EM.0.1.g

[B85] Turano K. A. Geruschat D. R. Baker F. H. Stahl J. W. Shapiro M. D. (2001). Direction of gaze while walking a simple route: persons with normal vision and persons with retinitis pigmentosa. *Optom. Vis. Sci.* 78 667–675. 10.1097/00006324-200109000-00012 11587201

[B86] Turano K. A. Yu D. Hao L. Hicks J. C. (2005). Optic-flow and egocentric-direction strategies in walking: central vs peripheral visual field. *Vis. Res.* 45 3117–3132. 10.1016/j.visres.2005.06.01 16084556

[B87] Velisavljević L. Elder J. H. (2008). Visual short-term memory for natural scenes: effects of eccentricity. *J. Vis.* 8 1–17. 10.1167/8.4.28 18484867

[B88] Võ M. L. H. Henderson J. M. (2011). Object–scene inconsistencies do not capture gaze: evidence from the flash-preview moving-window paradigm. *Atten. Percept. Psychophys.* 73 1742–1753. 10.3758/s13414-011-0150-6 21607814

[B89] Walther D. B. Caddigan E. Fei-Fei L. Beck D. M. (2009). Natural scene categories revealed in distributed patterns of activity in the human brain. *J. Neurosci.* 29 10573–10581. 10.1523/jneurosci.0559-09.2009 19710310 PMC2774133

[B90] Whitney D. Levi D. M. (2011). Visual crowding: a fundamental limit on conscious perception and object recognition. *Trends Cogn. Sci.* 15 160–168. 10.1016/j.tics.2011.02.005 21420894 PMC3070834

[B91] Wilkinson M. O. Anderson R. S. Bradley A. Thibos L. N. (2016). Neural bandwidth of veridical perception across the visual field. *J. Vis.* 16 1–17. 10.1167/16.2.1 PMC5833322 26824638

[B92] Williams L. J. (1989). Foveal load affects the functional field of view. *Hum. Perform.* 2 1–28. 10.1207/s15327043hup0201_1

[B93] Wilson H. R. Levi D. M. Maffei L. Rovamo J. DeValois R. (1990). “The perception of form: retina to striate cortex,” in *Visual Perception: The Neurophysiological Foundations*, eds Spillmann L. Werner J. S. (San Diego, CA: Academic Press, Inc), 231–272. 10.1016/b978-0-12-657675-7.50016-8

